# Child Social Exclusion Risk and Child Health Outcomes in Australia

**DOI:** 10.1371/journal.pone.0154536

**Published:** 2016-05-06

**Authors:** Itismita Mohanty, Martin Edvardsson, Annie Abello, Deanna Eldridge

**Affiliations:** 1 National Centre for Social and Economic Modelling, University of Canberra, Canberra, Australia; 2 Centre for Research and Action in Public Health, University of Canberra, Canberra, Australia; 3 Australian Institute of Health and Welfare, Canberra, Australia; TNO, NETHERLANDS

## Abstract

**Introduction:**

This paper studies the relationship between the risk of child social exclusion, as measured by the Child Social Exclusion (CSE) index and its individual domains, and child health outcomes at the small area level in Australia. The CSE index is Australia’s only national small-area index of the risk of child social exclusion. It includes five domains that capture different components of social exclusion: socio-economic background, education, connectedness, housing and health services.

**Methods:**

The paper used data from the National Centre for Social and Economic Modelling (NATSEM), University of Canberra for the CSE Index and its domains and two key Australian Institute of Health and Welfare (AIHW) data sources for the health outcome measures: the National Hospital Morbidity Database and the National Mortality Database.

**Results:**

The results show positive associations between rates of both of the negative health outcomes: potentially preventable hospitalisations (PPH) and avoidable deaths, and the overall risk of child social exclusion as well as with the index domains. This analysis at the small-area level can be used to identify and study areas with unexpectedly good or bad health outcomes relative to their estimated risk of child social exclusion. We show that children’s health outcomes are worse in remote parts of Australia than what would be expected solely based on the CSE index.

**Conclusions:**

The results of this study suggest that developing composite indices of the risk of child social exclusion can provide valuable guidance for local interventions and programs aimed at improving children’s health outcomes. They also indicate the importance of taking a small-area approach when conducting geographic modelling of disadvantage.

## 1. Introduction

Social exclusion is a broad concept used to describe social disadvantage and lack of resources, opportunity, participation and skills [1]. This paper considers the concept of social exclusion as it applies to children and their health outcomes. Childhood is crucial to our understanding of the determinants of disadvantage in society, and there has been a surge of interest in developing multidimensional measures of child wellbeing [2]. Child social exclusion has been defined as exclusion from: (1) Social activities (celebrations on special occasions, playgroups, swimming, holidays, school trips, etc); (2) Local services (library access, public transport, youth clubs, after-school clubs, nurseries and playgroups); and (3) School resources (teacher shortages, sharing school books, not enough computers at school, large class sizes, school buildings in disrepair) [3]. Recognising that children are very much dependent on their families, studies of child social exclusion usually include measures of family and dwelling characteristics rather than being focussed solely on child characteristics. Recent studies indicate that with few exceptions, children are at greater risk of living in poverty than the population as a whole [4–6]. There is evidence that lack of employment for parents and lack of educational opportunities for children are key determinants of child disadvantage [7].

Geographic analysis of child disadvantage and associated poor health outcomes is important from a policy perspective as it could highlight areas where local services are struggling to provide effective support, or identify how different underlying determinants of social disadvantage and health vary between areas [8]. This paper studies the link between child social exclusion and child health outcomes in Australia at the small-area level.

Significant research has been undertaken on child social exclusion both in Australia and internationally [9–13]. In Australia, Harding et al. (2009)[10], and Tanton et al. (2010)[12], developed an innovative approach to measure child social exclusion by identifying and combining different aspects of child disadvantage at a small-area level into the Child Social Exclusion Index (CSE Index). More recently, Abello et al (2012)[9] further developed the CSE Index. Drawing on the latest developments in child indicator research, they incorporated new data and grouped the individual indicators into domains that reflected key dimensions of child social exclusion before combining these into a composite index of CSE. The CSE Index defines child social exclusion based on parental, family, household and dwelling characteristics along the four widely identified dimensions of social exclusion (see [14]): (1) Consumption or lack of capacity to purchase goods and services; (2) production or inability to find employment; (3) political participation and (4) social interaction and family support. It is used to summarise the general risk of social exclusion faced by *all* children aged 0–15 years living within an area.

The CSE Index has demonstrated pronounced geographical variation with high social exclusion risk in rural and regional areas and in clusters in outer areas of Australia’s capital cities [9,10,12]. There is also much variation between states and terrories. New South Wales, Queensland and the Northern Territory have relatively high proportions of children at risk of social exclusion compared to the rest of Australia [9]. However, there are some aspects of potential disadvantage such as health that have not been included in the CSE index due to data limitations. The index includes a health services domain based on the number of GPs and dentists per 1000 persons in the small areas. However, service provider to population ratios may not be an accurate indicator of access to health services at the small area level as people travel between areas to access services [15]. The CSE index does not include any information on the health status of children or health care service delivery outcomes. It is not known how the index domains–including the health services domain, are related to health outcomes in children aged 0 to 15.

This study investigates how the CSE index and its domains, which represent key components of child social exclusion (socioeconomic background, education, connectedness, health services and housing), are related to child health outcomes in the form of potentially preventable hospitalisations and avoidable mortality at the small area level. It shows that areas with a high risk of social exclusion have worse health outcomes for children than other areas, but also that remote areas have worse health outcomes than what would be expected based on their CSE index scores alone (also see[16]).

This is a unique effort to explore the relationship between child health outcomes and the key components of child social exclusion in Australia. Similar relationships have been investigated in other countries with varying results [17–20]. In Canada, in a universal health insurance setting, Agha, et al. (2007)[17], found a large positive relationship between socioeconomic disadvantage and both hospitalisations for Ambulatory Care Sensitive (ACS) conditions and all-cause hospitalisations in children. Roos et al. (2005)[19], showed that ambulatory visits by physicians are more effective in preventing ACS hospitalisations for ACS conditions among relatively affluent individuals than among the less well off. However, Casanova et al. (1996)[18], could not identify any relationship between socio-demographic and primary-care factors associated with paediatric hospitalisation for ambulatory care sensitive conditions within a universal health care system in Spain. No such research has been conducted in Australia with the exception of Butler et al. (2013)[21], who found a positive association between risk of social exclusion and rates of ACS hospitalisations in 0–4 year olds across 79 Local Government Areas in the state of Victoria. No studies have examined how social exclusion at the small area-level relates to child health outcomes beyond early childhood years in Australia. While the early childhood years are prospectively the best time for intervention and hence mitigation of the longer term consequences of social deprivation on health [22], children are considered dependent and malleable until they reach 16 years. While cognitive capabilities are malleable in childhood before age 10, non-cognitive capabilities are malleable until later ages [23]. This brings the focus of this study on children aged 0 to 15 years. The cut-off age of 15 years for dependent children is standard Australian [24–26] and international practice.

In this study, routinely collected data on potentially preventable hospitalisations (PPHs) and avoidable deaths compiled by the Australian Institute of Health and Welfare (AIHW) are used as measures of health outcomes at the small area level.

Potentially preventable hospitalisations, also referred to in the literature as hospitalisations for ambulatory care sensitive conditions (ACSCs), are hospitalisations that could have potentially been prevented through the provision of appropriate non-hospital health services such as primary health care interventions and early disease management [27,28]. PPHs can be classified into three main types: Vaccine-preventable; chronic; and acute conditions [28]. Research has shown that poor access to primary health care is strongly related to higher rates of PPHs [29–33]. However, to the extent that PPHs are associated with access to health services, it remains that some events that are classified as PPH will not always be avoidable. The literature suggests that PPH indicators should therefore be treated as a set of conditions rather than being examined as individual disease specific outcomes. Further, it would be more appropriate to adjust PPHs for demographic factors such as the age distribution in a particular area so that the overall rates would not be affected by, for example, higher rates of risk factors generally experienced by older people [29]. PPHs are important not only because they represent poor health outcomes that can be avoided but also because of the ever growing need for a more sustainable and cost effective health care system. A shift in policy focus from the acute to the primary health care sector and to prevention of hospitalisations is one way in which this could be achieved.

Avoidable deaths are deaths from certain conditions that are considered avoidable given timely and effective health care. They also include deaths amenable to legal measures, such as traffic safety (for example, speed limits and use of seat belts and motorcycle helmets) [34].

## 2. Data and Methods

### 2.1 Data

The analysis presented in this paper relies on the National Centre for Social and Economic Modelling (NATSEM), University of Canberra for the CSE Index and its domains and two key Australian Institute of Health and Welfare data sources for the health outcome measures: the National Hospital Morbidity Database and the National Mortality Database, which contain records of hospital separations and deaths occurring in Australia (see [16]).

The geographical unit of analysis is the Australian Bureau of Statistic’s Statistical Local Area (SLA) as of 2006 (see Harding et al. (2009)[10], for a full description of the rationale for our choice of this geographical unit). While very commonly used for geographical analysis in Australia, it is important to note that SLA populations differ in size. As more populous areas are more likely to cover heterogeneous populations, smaller pockets of disadvantage within such areas may be undetectable in the results. This is less likely to occur in less populous SLAs as they are more likely to have relatively homogenous populations. In order to address this issue, we aggregated SLAs in the Australian Capital Territory and Brisbane (the two regions where the SLAs are most consistently comparatively small compared with other SLAs across Australia) such that their populations and relative heterogeneity would more closely match those of SLAs across the remainder of Australia.

Calculated for individual SLAs, the CSE Index is largely based on data from the 2006 Australian Census of Population and Housing (see section 3.1.1 below). SLAs that had fewer than 30 resident children aged 0–15 years or an 80 per cent or higher non-response rate for any census variable included in the social exclusion index were excluded from analysis. A total of 35 small areas were excluded due to low child population and an additional 3 small areas that had both low population and low response. With those SLAs in the ACT and Brisbane aggregated to larger areas as previously described, this resulted in a total of 1154 small areas for use in creating the CSE Index. Many of the small areas that were excluded from the analysis were in the remote areas of northern and central Australia, including many remote Indigenous communities.

#### 2.1.1 CSE Index and domains

The CSE Index is a small area measure of social exclusion risk for children in Australia. Data were sourced from the 2006 Australian Census of Population and Housing, supplemented with 2009 National Assessment Program–Literacy and Numeracy (NAPLAN) and 2009 Australian Early Development Index (AEDI) scores on educational outcomes. The CSE Index includes five domains that reflect key dimensions of child wellbeing. The indicators chosen to measure child social exclusion are listed in Table 1, classified by domain group.

**Table 1:** CSE index domains and indicators.

Domain	Sub-domain	Indicator
Socio-economic	Sole parent family	Proportion of dependent children aged 0–15 in a single parent family
	Bottom income quintile	Proportion of dependent children aged 0–15 in a household with income in the bottom quintile of equivalent gross household income among all households in Australia
	No parent in paid work	Proportion of dependent children aged 0–15 in a family where no parent is working
Education	No family member completed Year 12	Proportion of dependent children aged 0–15 with no one in the family having completed Year 12
	NAPLAN children in Year 5*	A statistical proportion based on the Year 5 National Assessment Program–Literacy and Numeracy (NAPLAN) average reading score and average numeracy score, divided by the national average (2009)
	AEDI children in first year of school	Proportion of children who scored below the 10^th^ percentile on the AEDI (2009)
Connectedness	No internet at home	Proportion of dependent children aged 0–15 living in a household with no internet access
	No parent doing voluntary work	Proportion of dependent children aged 0–15 in a family where no parent is doing voluntary work
	No motor vehicle	Proportion of dependent children aged 0–15 living in a household with no motor vehicle
Housing	High rent and low income (30/40 rule)	Proportion of dependent children aged 0–15 living in a household where rent is 30% or more of household income, and in bottom two quintiles of equivalent gross household income
	Overcrowding	Proportion of dependent children aged 0–15 living in a household that needs one or more additional bedrooms
Health service access	Ratio of GPs*	A statistical proportion based on the number of General Practitioners (GPs) divided by the total population ('000s)
	Ratio of dentists*	A statistical proportion based on the number of dentists divided by the total population ('000s)

* These metrics were converted to proportions so as to be comparable with all other variables.

Source: Australian Census of Population and Housing 2006, ACARA 2009, AEDI 2009.

The CSE index is continually being updated and its construction is outside the scope of this paper. It was first generated using 2001 Census data. Thereafter it was updated using 2006 Census data, then 2006 Census data were augmented with 2009 NAPLAN and AEDI data. NAPLAN first occurred in 2008, but AEDI data were first available for 2009. It therefore made sense to use the NAPLAN data for the same year.

Indicators and domains were constructed to include dependent children aged 0–15 years. The AIHW health measures are calculated for children aged 0–14 years.This slight difference is due to alternative coding conventions and is not expected to have a substantial effect on the results presented.

Nearly all the indicators are expressed in the form of proportions, with larger values indicating higher risk of social exclusion. The only exceptions were the GP and dentist ratios and the NAPLAN index ratios, where lower values initially indicated higher risk of social exclusion. To be consistent with the other variables, these ratios were transformed such that higher values indicated a higher risk of social exclusion.

Principal components analysis (PCA) was used to summarise the variables within the Socioeconomic, Education, Connectedness and Health Service domains into a single score for each domain. PCA has been used in Australia and other countries to create summary indices from a number of correlated variables (see [35,36]). Because of the weak correlation between the two housing variables (high renting cost and overcrowding), PCA was not used for the Housing domain. The arithmetic mean of these variables was taken to constitute the domain score for Housing.

As the five domain indices had different units of measurement, these were transformed into comparable figures using an exponential transformation of ranks, following the formula described in Noble et al. (2004)[37], and Bradshaw et al. (2009)[38]. The transformation used is as follows: for any small area to denote its rank on the index, scaled to the range [0 to 1], by R (with R = 1/N for the least deprived, and R = N/N, i.e., R = 1, for the most deprived, where N = the total number of small areas). The transformed index, X say, is X = -23*log {1—R*[1—exp(-100/23)]} where log denotes natural logarithm and exp the exponential or antilog transformation.Then the arithmetic mean of the five domain indices was taken to form the composite CSE index. Finally, in order to produce results that would be easily interpretable, as well as to address the issue of unequal population numbers in small areas, the final index scores were used to calculate child-population weighted quintiles of child social exclusion risk. The results are presented using these quintiles, with the lowest quintile representing the highest risk of social exclusion and higher quintiles representing lower risk of social exclusion. The bottom social exclusion quintile thus represents the *20 per cent of children* (rather than 20 per cent of small areas) facing the highest risk of being socially excluded. Further details on the construction of the CSE index can be found in Abello et al.(2012)[9].

#### 2.1.2 Health measures. 2.1.2.1 Potentially preventable hospitalisations

Potentially Preventable Hospitalisations (PPH), or ambulatory care sensitive conditions (ACSCs), include hospitalisations thought to be avoidable with the application of public health interventions and early disease management. In Australia PPH are considered a key performance indicator with high rates of PPH seen to provide indirect evidence of problems with patient access to primary health care, inadequate skills and resources, or disconnection with specialist services [21]. We used the same selection of ICD-10-AM codes (the Australian modification of the 10^th^ revision of the World Health Organisation’s International Classification of Diseases) to define potentially preventable hospitalisations(PPH) in this study as the selection used for reporting in *Australian Hospital Statistics 2010–11*[28].This selection of ICD-10-AM codes is also consistent with the selection used by the *Victorian Ambulatory Care Sensitive Conditions Study* [39] and the *Atlas of Avoidable Hospitalisations in Australia*: *ambulatory care-sensitive conditions* [27]. Using this selection of ICD-10-AM codes, we extracted records of PPH from the AIHW’s National Hospital Morbidity Database (see http://www.aihw.gov.au/hospitals/australian-hospital-statistics/).

We were able to calculate average annual rates of potentially preventable hospitalisations (PPH) (number per 1000, 0–14 year olds) for 1112 of the 1154 small areas that were used when the CSE Index was created. To reduce the influence of stochastic year-to-year variation in PPH rates in the small areas, we used PPH data from three financial years (2006–07, 2007–08, 2008–09), starting with the year when the 2006 Census data on which the CSE Index is mostly based were collected and ending with the year when the additional data that were incorporated into the updated CSE Index were collected (2009). The December population sizes of all small areas for 2006–07, 2007–08 and 2008–09 were necessary for the rate calculations. The Australian Bureau of Statistics’ estimated resident populations (ERP) at June 2006, 2007, 2008 and 2009 were used to estimate these populations. The December population for each small area was estimated by taking the average of the preceding and subsequent June ERPs.

PPH rate varies with age in 0–14 year olds. Therefore, to reduce the effect of variation in age structure between the small areas, we age-standardised the PPH rates such that in all small areas, the proportional contributions to the overall rate for 0–14 year olds from the age groups 0–4, 5–9 and 10–14 years were identical to those of the same age groups in the Australian standard population of 2001[40].

#### 2.1.2.2 Avoidable mortality

Avoidable mortality is another key measure of the performance of the healthcare system and the effectiveness of policy measures aimed at reducing fatal accidents [41]. To define avoidable mortality in this study, we used the ICD-10 codes that were used for the 2012 cycle of reporting of the Australian National Healthcare Agreement’s progress indicator PI 20 (including both treatable and preventable causes of death) for avoidable deaths [34]. We extracted records of avoidable deaths from AIHW’s National Mortality Database (see http://www.aihw.gov.au/deaths/aihw-deaths-data/).

We calculated rates of avoidable deaths for 2007 for 0–14 year olds in all of the 1154 small areas that were used when the CSE Index was created (data on avoidable deaths from one year only were sufficient for our analysis–see below). Areas with no recorded avoidable deaths were assumed to have had no avoidable deaths. The population sizes of the small areas were derived from the Australian Bureau of Statistics’ Estimated Resident Population at June 2007.

### 2.2 Methods

#### 2.2.1 Potentially preventable hospitalisations

To examine whether areas that have a high risk of social exclusion, as indicated by the CSE index, also are disadvantaged by high rates of PPH, we calculated the correlations between the index (and its domains) and PPH rates. We present Pearson correlation coefficients weighted by the sizes of the population of 0–15 year olds of the small areas using raw index scores in Table 2 (Spearman rank correlations produced very similar results).

**Table 2:** Pearson correlation between rates of PPH and the CSE Index and its domains.

	Pearson correlation
**CSE index score**	0.40***
**Socio-economic domain**	0.40***
**Education domain**	0.38***
**Connectedness domain**	0.38***
**Housing domain**	0.28***
**Health Services domain**	0.06^ns^

*** p<0.001

** p<0.05

^ns^ Not statistically significant.

To further investigate the association between PPH rate and risk of social exclusion, we ranked the small areas by PPH rate and divided them into quintiles such that each quintile included 20% of the total population of children in all areas. The first quintile represents small areas with the highest PPH rates (worst health care outcomes) and the fifth quintile represents small areas with the lowest PPH rates (best health care outcomes). We also created quintiles based on the CSE index and all its individual domains in the same way. The PPH rate quintiles, the CSE index quintiles and the quintiles based on all individual index domains were all created using the population of 0–15 year olds that was used when the CSE index was constructed. Tables 3–8 show the proportion of children in each PPH rate quintile cross-tabulated by each CSE index or domain quintile. The diagonals indicate the proportion of children whose PPH quintile is the same as the CSE index or domain quintile. That is, the diagonal cells include the population of the areas where there is a concordance in the disadvantage estimated from both measures of disadvantage. The total proportion of children on the diagonal (and adjacent highlighted cells) is summarised at the bottom of each table. In general the higher these proportions, the greater the association between PPH and the measure of interest.

**Table 3:** Proportion of children aged 0–14: PPH quintile by CSE index quintile.

PPH quintile	CSE index quintile					Total
(age-standardised)	1	2	3	4	5	
	%	%	%	%	%	%
**1** Highest PPH	**7.3**	6.2	3.5	2.0	1.1	20.0
**2** Mid-high PPH	5.7	**4.6**	4.0	3.9	1.9	20.1
**3** Middle group	2.3	2.6	**5.1**	5.2	5.1	20.2
**4** Mid-low PPH	2.9	4.5	3.5	**4.2**	4.7	19.8
**5** Lowest PPH	1.6	2.2	3.9	4.9	**7.3**	19.9
Total	19.7	20.1	20.0	20.1	20.1	100.0

Diagonal: 28.5% of all children; Diagonal and adjacent cells: 65.2% of all children.

**Table 4:** Proportion of children aged 0–14: PPH quintile by Socio-economic domain quintile.

PPH quintile	Socio-economic domain quintile					Total
(age-standardised)	1	2	3	4	5	
	%	%	%	%	%	%
**1** Highest PPH	**8.3**	6.1	3.4	1.0	1.2	20.0
**2** Mid-high PPH	4.6	**5.8**	4.3	3.4	2.1	20.1
**3** Middle group	1.9	2.5	**6.1**	3.7	6.0	20.2
**4** Mid-low PPH	3.3	3.5	3.0	**5.5**	4.6	19.8
**5** Lowest PPH	1.8	2.2	3.3	6.5	**6.2**	19.9
Total	19.8	20.1	20.0	20.1	20.0	100.0

Diagonal: 31.8% of children; Diagonal and adjacent cells: 67.0% of children.

**Table 5:** Proportion of children aged 0–14: PPH quintile by Education domain quintile.

PPH quintile	Education domain quintile					Total
(age-standardised)	1	2	3	4	5	
	%	%	%	%	%	%
**1** Highest PPH	**7.3**	6.0	3.7	2.7	0.4	20.0
**2** Mid-high PPH	4.2	**4.3**	4.7	4.7	2.2	20.1
**3** Middle group	3.0	3.5	**4.1**	4.5	5.1	20.2
**4** Mid-low PPH	2.8	4.3	3.9	**2.8**	6.0	19.8
**5** Lowest PPH	2.1	2.3	3.6	5.5	**6.4**	19.9
Total	19.5	20.3	19.9	20.3	20.1	100.0

Diagonal: 24.7% of children; Diagonal and adjacent cells: 63.0% of children.

**Table 6:** Proportion of children aged 0–14: PPH quintile by Connectedness domain quintile.

PPH quintile	Connectedness domain quintile					Total
(age-standardised)	1	2	3	4	5	
	%	%	%	%	%	%
**1** Highest PPH	**7.2**	5.5	3.0	2.5	1.8	20.0
**2** Mid-high PPH	5.9	**4.7**	4.3	2.8	2.4	20.1
**3** Middle group	2.1	4.4	**5.2**	3.9	4.6	20.2
**4** Mid-low PPH	3.1	4.0	3.5	**4.6**	4.7	19.8
**5** Lowest PPH	1.7	1.3	4.2	6.2	**6.6**	19.9
Total	19.9	19.9	20.2	20.0	20.0	100.0

Diagonal: 28.1% of children; Diagonal and adjacent cells: 66.6% of children.

**Table 7:** Proportion of children aged 0–14: PPH quintile by Housing domain quintile.

PPH quintile	Housing domain quintile					Total
(age-standardised)	1	2	3	4	5	
	%	%	%	%	%	%
**1** Highest PPH	**5.5**	6.0	4.2	2.1	2.3	20.0
**2** Mid-high PPH	6.8	**4.0**	3.9	3.6	1.8	20.1
**3** Middle group	1.7	3.7	**3.9**	5.9	5.0	20.2
**4** Mid-low PPH	3.5	4.2	4.4	**4.2**	3.5	19.8
**5** Lowest PPH	2.2	2.0	4.0	4.3	**7.5**	19.9
Total	19.6	19.9	20.3	20.1	20.0	100.0

Diagonal: 25.1% of children; Diagonal and adjacent cells: 63.5% of children.

**Table 8:** Proportion of children aged 0–14: PPH quintile by Health services domain quintile.

PPH quintile	Health services domain quintile					Total
(age-standardised)	1	2	3	4	5	
	%	%	%	%	%	%
**1** Highest PPH	**3.9**	4.1	4.6	4.7	2.7	20.0
**2** Mid-high PPH	3.3	**3.8**	4.7	3.7	4.6	20.1
**3** Middle group	4.1	4.8	**3.0**	5.5	2.7	20.2
**4** Mid-low PPH	3.6	4.4	3.4	**1.9**	6.5	19.8
**5** Lowest PPH	4.8	2.7	4.4	4.4	**3.6**	19.9
Total	19.8	19.9	20.0	20.2	20.1	100.0

Diagonal: 16.3% of children; Diagonal and adjacent cells: 52.9% of children.

One-way analysis of variance (ANOVA) was used to test for differences in mean PPH rate between areas in the different quintile groups. The Kruskal-Wallis test and Welch’s t-test were used to complement the results of the ANOVA as there was substantial variation in PPH rate variance between quintiles (see also section 4.1).

**2.2.1.1 Areas with unexpectedly high or low rates of PPH:** We used residuals from linear regression models with area PPH rate as the response variable and area CSE index score as the explanatory variable to identify areas with unexpectedly high or low rates of PPH given their risk of social exclusion. Models including the index scores, the square of the index scores and the cube of the index scores were explored in order to find the model that was best able to predict an area’s PPH rate based on its CSE index score. Given that PPH rates differed the most between the two quintiles with the highest risk of child social exclusion, the best model was likely to include the square or the cube of the index scores. Following stepwise elimination, the best model that included only significant explanatory variables was one with only the square of the CSE index: PPH rate = 0.0071 * CSE index^2^ + 21.74; (p < 0.001, r^2^ = 0.22.). Based on this model, we calculated residual PPH rates for all areas.

The residual PPH rate for an area is equal to the deviation of the PPH rate of that area from the rate predicted by the model based on the risk of social exclusion in that area as measured by the CSE index. Residuals can therefore be used to identify areas with unexpectedly high or low rates of PPH, given their risk of social exclusion, and to examine what characterises these areas. Using residuals from regression models is a straightforward way to quantify how much rates of individual areas deviate from what would be expected based on the CSE index without relying on any preconceived ideas about the relationship between CSE and PPH. Having the residuals makes it easy to analyse patterns and test hypotheses about what drives deviations from the expected CSE index scores.

We used the residuals to test whether areas with a certain risk of CSE had consistent rates of PPH across areas with different degrees of remoteness. All areas were placed in one of the Australian Bureau of Statistics’ five remoteness categories (*Major cities*, *Inner regional*, *Outer regional*, *Remote* and *Very remote*), which are based on the remoteness of a location from services provided by large towns or cities. Areas that fell within more than one remoteness category were placed in the category where the majority of the population in that area lived.

#### 2.2.2 Avoidable mortality

Annual rates of avoidable deaths were too low to enable reliable estimates of the rates of individual small areas even if data from several years were aggregated. For each year, there were many areas with no deaths recorded and most of the rest of the areas had one to a few deaths. Therefore, we focused our analysis on comparing rates of avoidable mortality across index and domain quintiles. There were enough avoidable deaths in one year for this type of analysis.

We calculated the overall rates of avoidable deaths among 0–14 year olds of all areas in each quintile (based on deaths registered in 2007 and the Australian Bureau of Statistics’ estimated resident population of 0–14 year olds) and used these rates to estimate the number of deaths of all areas in each quintile among 0–15 year olds (the population used to create the index and domain quintiles). We then used chi-square tests to assess whether rates differed significantly between index and domain quintiles.

Only p values that are smaller than 0.05 are referred to as significant in this paper.

## 3. Results

### 3.1 Potentially preventable hospitalisations

Table 2 shows Pearson correlation coefficients weighted by the sizes of the population of 0–15 year olds of the small areas using raw index scores and PPH rates.There is a moderately strong and significant positive correlation between rates of PPH and the CSE index scores (Pearson correlation weighted by the population sizes of the small areas: r = 0.40, p<0.001). This means that children in areas with a relatively high risk of social exclusion tended to have relatively poor health outcomes in terms of potentially preventable hospitalisations. We found similar results (moderately strong and significant positive correlations) between PPH rates and the socio-economic, education, connectedness and housing domains (Table 2). However, there is only a weak and not significant correlation between PPH rates and the health services domain.

Table 3 shows the proportion of children in PPH rate quintiles cross-tabulated with the CSE index quintiles. It reveals 28.5 per cent of children have the same quintile rank on the CSE index and PPH rates (diagonals sum to 28.5 per cent) and that 65.2 per cent of children have the same or close to the same quintile rank on the CSE index and PPH rates (diagonals and adjacent cells sum to 65.2 per cent). This is consistent with the moderate degree of correlation between PPH and the composite CSE index in Table 2.

Table 4 to Table 8 show the proportion of children in PPH rate quintiles cross-tabulated with the quintiles of each CSE index domain. Again, the numbers in these tables are consistent with the moderate degree of correlation between PPH rate and four out of the five CSE index domains. With the proportion of children along the diagonals summing to only 16 per cent, the Health services domain clearly has the weakest association with PPH rate.

**Fig 1:**
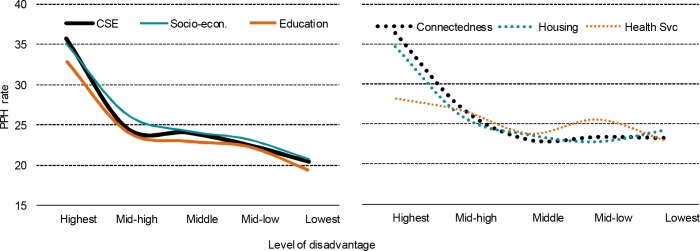
PPH rates by levels of disadvantage on CSE index and domains.

Table 9 shows the mean PPH rates of quintile groups of the CSE index. There are significant differences in PPH rates between areas in the different quintile groups (one-way Analysis of Variance (ANOVA): F_4, 1107_ = 38.01, p < 0.001). Note that the high variance in PPH rates observed in the most disadvantaged quintile relative to the variance in the other quintiles would be expected to make the one-way ANOVA more conservative as the most disadvantaged quintile included the largest number of areas (see e.g. [42]). Also, the significant differences between quintiles was confirmed by a non-parametric Kruskal-Wallis test (χ^2^ = 144.25, df = 4, p < 0.001), which is less sensitive to deviations from equal variances across groups than the ANOVA. As expected, there is a clear gradient in PPH rates with small areas in the bottom quintile (highest social exclusion risk) having the highest average PPH rates and areas in the top quintile (lowest social exclusion risk) having the lowest rates. The biggest difference occurred between quintiles with the highest and second highest risk of social exclusion. Differences in average rates were much smaller between the other quintiles. This is evident in Fig 1 (left panel), which plots the average PPH rate for each CSE quintile group from most disadvantaged, to least disadvantaged. For example, Table 9 shows an annual average of 35.7 potentially preventable hospitalisations per 1,000 children for quintile 1 (CSE quintile with the highest risk of social exclusion), and rates ranging from 24.6 for quintile 2 to 20.4 for quintile 5. Welch’s t-tests, which are not sensitive to unequal variances between groups, found significant differences in PPH rates between areas in the first index quintile (highest risk of social exclusion) and areas in all other quintiles (1 vs 2: t_395.33_ = 6.77, 1 vs 3: t_385.36_ = 7.18, 1 vs 4: t_374.7_ = 8.19, 1 vs 5: t_346.98_ = 9.68, all p < 0.001), between the second and fourth quintiles (t_444.54_ = 2.17, p = 0.03), between the second and fifth quintiles (t_359.38_ = 4.62, p < 0.001), between the third and fifth quintiles (t_374.76_ = 4.16, p < 0.001) and between the fourth and fifth quintiles (t_342.35_ = 2.46, p = 0.01).

**Table 9:** PPH rates by quintile of CSE index.

CSE quintile group	Mean	Std. dev.	Minimum	Maximum
**1** Highest risk of social exclusion	35.7	24.6	4.4	193.6
**2**	24.6	10.8	7.1	83.2
**3** Intermediate risk	24.0	10.7	5.4	108.5
**4**	22.5	9.5	3.5	75.3
**5** Lowest risk of social exclusion	20.4	6.4	8.9	45.6

The same pattern—average rate of PPH declining across quintiles and being much higher in the first quintile than in all other quintiles—is found when comparing the socio-economic and education domains quintiles (see Fig 1).

The standard deviations of Table 9 show that variation between areas in PPH rates is the greatest in the most disadvantage quintiles of the CSE Index and all its domains. While the most disadvantaged quintile has the highest average PPH rates, there are both areas in this quintile with very low PPH rates and areas with very high rates. Furthermore, the high average rate is not caused by a small number of areas with very high rates. There are 41 areas with a PPH rate above 50 (more than twice the average of the second quintile) in the most disadvantaged quintile and 8 areas with a rate above 100.

Turning to the connectedness, housing and health services domains, the gradient in PPH rates by quintile is less clear. While there is still a large difference between the first quintile and all other quintiles, there is no consistent pattern across the other quintiles (Fig 1, right panel).

#### 3.1.1 Areas with unexpectedly high or low rates of PPH

Table 10 shows the residual PPH rates by remoteness estimated from the linear regression models with area PPH rate and the square of the CSE index score. It is evident that small areas in *Major cities*, *Inner regional* areas and *Outer regional* areas have PPH rates close to or just below what our model predicts based on their risk of social exclusion (the mean residual PPH rate is close to 0). However, *Remote* and *Very remote* areas have PPH rates that are higher on average than what the model predicts (high residual PPH rates). In other words, areas that are *Remote* or *Very remote* generally have PPH rates that are higher than what would be expected solely based on their CSE risk. This pattern is confirmed by an ANOVA that showed a significant association between remoteness category and residual PPH rates (F_4, 1107_ = 16.36, p < 0.001). A post-hoc Tukey test revealed that areas that are *Remote* or *Very remote* have residual PPH rates that are significantly higher on average than all other areas (all p < 0.05). There are no significant differences between *Remote* and *Very remote* areas or among areas classified as *Major cities*, *Inner regional* or *Outer regional*.

**Table 10:** Residual PPH rate by remoteness.

		Analysis Variable: Residual PPH rate			
Remoteness	Mean	Std. Dev.	Minimum	Maximum	N
Major cities	-2.4	6.0	-16.4	47.7	383
Inner regional	-0.5	10.3	-20.4	90.0	263
Outer regional	-0.9	10.9	-20.5	39.7	293
Remote	8.3	25.6	-47.2	140.0	82
Very remote	6.8	29.8	-40.7	167.4	91

Note: Areas with lower PPH rates than what the model predicts based on their risk of social exclusion have negative residuals and areas with higher than predicted rates have positive residuals.

*Remote* and *Very remote* areas have a higher proportion of Indigenous people of Australian Aboriginal and/or Torres Strait Islander descent than other parts of Australia. However, because many remote Indigenous communities are located in areas that had to be excluded from the CSE index (see section 3.1), how representative the residual PPH rates of *Remote* and *Very remote* areas are for Indigenous people in remote communites is unclear. Indigenous Australians generally have poorer health outcomes, including higher PPH rates, than other Australians and tend to have worse access to primary health care across all remoteness categories [15]. As the CSE index does not account for Indigenous status, we were not able to investigate what role child social exclusion plays in the health outcomes of Indigenous children.

### 3.2 Avoidable mortality

Table 11 and Table 12 presents contingency tables with total number of estimated deaths and survivors (children who did not die because of avoidable causes–includes children who died because of non-avoidable causes) for all areas in each quintile of the composite index and its individual domains in 2007 (year of death registration). These tables give an overview of how rates of avoidable deaths vary between areas in the different index or domain quintiles. Chi-square tests based on these tables were used to test whether the variation seen is significant.

**Table 11:** Total number of estimated avoidable deaths and survivors in each quintile of the composite CSE Index and the Socioeconomic and Education domains in 2007 (year of registration).

Quintile of disadvantage	Composite CSE Index		Socioeconomic domain		Education domain	
	Avoidable deaths	Population	Avoidable deaths	Population	Avoidable deaths	Population
1 Highest	240	747511	229	750623	235	738789
2 Mid-high	175	759398	191	757969	164	766628
3 Middle	189	752010	167	752355	188	749371
4 Mid-low	163	756553	164	756258	153	762797
5 Lowest	114	757980	130	756247	141	755866
Total	881	3773452	881	3773452	881	3773451
Chi-square	χ^2^ = 49.9 DF = 4	p <0.001	χ ^2^ = 31.2 DF = 4	p < 0.001	χ ^2^ = 35.6, DF = 4	p < 0.001

**Table 12:** Total number of estimated avoidable deaths and survivors in each quintile of the Connectedness, Housing and Health services domains in 2007 (year of registration).

Quintile of disadvantage	Connectedness domain		Housing domain		Health services domain	
	Avoidable deaths	Population	Avoidable deaths	Population	Avoidable deaths	Population
1 Highest	215	752973	218	741278	195	751604
2 Mid-high	206	749644	211	753022	166	749686
3 Middle	174	760712	189	765405	159	755523
4 Mid-low	156	754042	131	757058	166	760827
5 Lowest	131	756081	132	756688	195	755811
Total	882	3773452	881	3773451	881	3773451
Chi-square	χ ^2^ = 28.3 DF = 4	p <0.001	χ ^2^ = 42.5 DF = 4	p < 0.001	χ ^2^ = 7.1 DF = 4	p = 0.13

**Fig 2:**
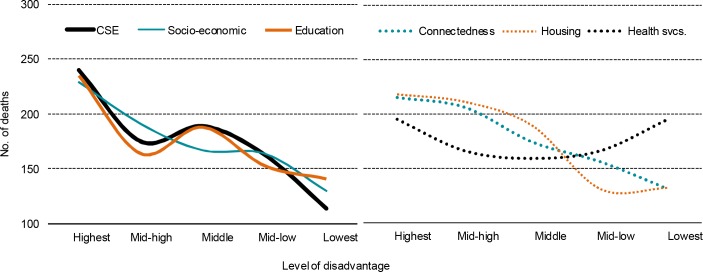
Estimated no. of deaths out of a population of approximately 75,000 in each quintile of the composite CSE index and its domains, 2007.

Rates of avoidable deaths are more than twice as high in areas in the first index quintile compared with areas in the fifth quintile (Table 11, Fig 2). This results in more than 100 extra annual deaths among the 20% of children who lived in areas with the highest risk of social exclusion compared with the 20% who lived in areas with lowest risk of social exclusion. The individual index domains show a similar pattern. The distribution of avoidable deaths across quintiles is significantly different from what would be expected if risks of avoidable deaths were the same in all quintiles. This is the case for all domains except the health services domain, which does not seem to have a strong association with avoidable deaths.

## 4. Discussion

This study found an association between the CSE index and child health outcomes. Children aged 0–14 years living in areas with a high risk of child social exclusion have higher rates of both potentially preventable hospitalisations and avoidable deaths. An earlier study by Butler et al. (2013)[21], found a similar association between the CSE index and PPH rates among 0–4 year olds in the Australian state of Victoria. These results suggest that developing composite indices of the risk of child social exclusion can provide valuable guidance for local interventions and programs aimed at improving children’s health outcomes. They also indicate the importance of taking a small-area approach when conducting geographic modelling of disadvantage.

In this study, similar associations with children’s health outcomes are found for the composite CSE index and its socio-economic, education, connectedness, and housing domains. The only CSE index domain that does not show a clear association with child health outcomes is the health services domain. Access to health services varies greatly across Australia and is associated with geographic variation in health outcomes [15]. The lack of association between the health services domain and children’s health outcomes therefore suggests that the health services domain needs to be developed further. Service to population ratios in the small areas used to calculate the CSE index may often not give an accurate view of geographic variation in access to health services. In many cases, people can easily travel to access health services outside the area they reside in. Recent research has shown that a geospatial index of access to health services relative to need that takes into account that people can travel to nearby areas to access health care can provide a more accurate view of variation in access to health care at the small area level [15].

Butler et al. (2013)[21], found a relatively even increase in rates of PPH among 0–4 year olds as the risk of child social exclusion increased across the five index quintiles. In this study, the difference in PPH rates is much greater between the quintiles representing the highest and the second highest risk of child social exclusion than between any other two consecutive quintiles. This difference could be caused by the association between the risk of child social exclusion and PPH rates being somewhat different in 0–4 year olds and 0–14 year olds. However, when *Remote* and V*ery remote* areas were excluded from the analysis in this study the change in PPH rates became more even across quintiles. The state of Victoria, where all areas included in the study by Butler et al. (2013)[21], were located, has no *Very remote* and few *Remote* areas.

Of course, many factors that are not captured by the CSE index influence children’s health outcomes. This study found that children living in *Remote* and *Very remote* areas often experienced higher PPH rates than what would be expected based on the CSE index scores of these areas. It appears that remoteness, as defined by the Australian Bureau of Statistics, is associated with children’s health outcomes in a way that is, at least partly, independent of social exclusion as measured by the CSE index. Further investigation of areas where children have unexpectedly high rates of PPH may reveal additional factors that are important to children’s health outcomes.

## 5. Conclusion

This study found similar associations between children’s health outcomes and the composite CSE index and its socio-economic, education, connectedness, and housing domains. The lack of association between the health services domain and children’s health outcomes suggests that this domain needs to be revisited. The differential impact of remoteness on children’s health outcomes could also be the subject of further study.
